# Comparison of subspecialty major surgical volume in the United States during the COVID-19 pandemic

**DOI:** 10.1002/lary.30066

**Published:** 2022-02-17

**Authors:** Anirudh Saraswathula, Ernie Shippey, Lee Ann Sprankle, Allen Kachalia, Redonda G. Miller, Christine G. Gourin, C. Matthew Stewart

**Affiliations:** Department of Otolaryngology-Head and Neck Surgery, Johns Hopkins University School of Medicine, Baltimore, Maryland, USA; Vizient^®^ Inc, Irving, Texas, USA; Department of Otolaryngology-Head and Neck Surgery, Johns Hopkins University School of Medicine, Baltimore, Maryland, USA; Division of General Medicine, Department of Medicine, Johns Hopkins School of Medicine, Baltimore, Maryland, USA; Armstrong Institute for Patient Safety and Quality, Johns Hopkins Medicine, Baltimore, Maryland, USA; Johns Hopkins Medicine, Baltimore, Maryland, USA; Department of Otolaryngology-Head and Neck Surgery, Johns Hopkins University School of Medicine, Baltimore, Maryland, USA; Department of Otolaryngology-Head and Neck Surgery, Johns Hopkins University School of Medicine, Baltimore, Maryland, USA; Armstrong Institute for Patient Safety and Quality, Johns Hopkins Medicine, Baltimore, Maryland, USA

## INTRODUCTION

The ongoing COVID-19 pandemic has profoundly impacted the provision of surgical care.^1,2^ We have previously reported that outpatient and inpatient otolaryngology surgical volume dropped over 80% and 40%, respectively, at the onset of the pandemic.^3^ We sought to describe the pandemic’s impact on otolaryngology subspecialty case volume and in comparison to other specialties.

We queried surgical volume and specialty from the Vizient Clinical Data Base™ (CDB) (Vizient, Inc. Irving, TX) from January 1, 2019, to March 31, 2021 (Supplementary Methods in the online version of this article). A case volume-weighted specialty interruption index was calculated for each specialty as the percentage of months with volume interruptions, defined as a > 30% decrease in volume for that specialty at that hospital system in that month compared to 2019 volume. Logistic regression was used to analyze outpatient surgical volume compared with 2019, adjusting for bed size, academic medical center (AMC) status, region, COVID-19 census, and average inpatient Centers for Disease Control Social Vulnerability Index (SVI), a composite metric of socioeconomic status. The 100 most common inpatient and outpatient otolaryngology codes were also categorized by subspecialty for more granular analysis. Data were analyzed using SAS v7.1.5 (SAS Institute Inc., Cary, North Carolina) and R v4.1.0 (R Foundation for Statistical Computing, Vienna, Austria). This study was approved as exempt by the Johns Hopkins University School of Medicine institutional review board.

## DISCUSSION

**Fig. 1.**
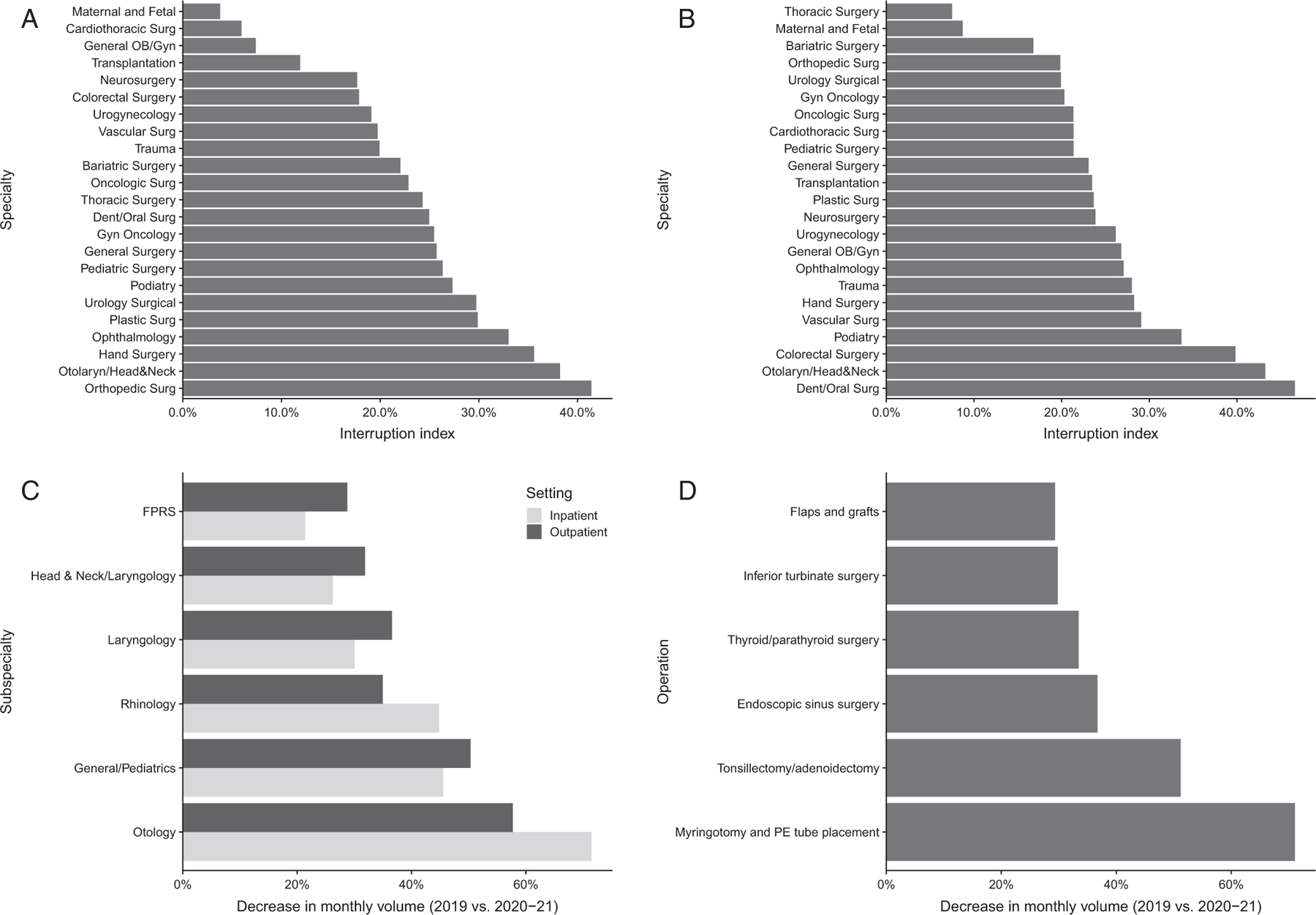
Interruption of major surgical case volume in 2020–2021 by surgical specialty for A) inpatient and B) outpatient surgery; decreases in monthly case volumes from 2019 to 2020–2021 C) by subspecialty of otolaryngology and D) in the most common outpatient otolaryngologic procedures. See Supplementary Methods in the online version of this article for calculation of interruption index, weighted by 2019 surgical volume. (FPRS: facial plastic and reconstructive surgery).

The characteristics of 299 hospital systems that comprised this study are shown in Supplementary Table 1 in the online version of this article. Inpatient surgical volumes (Figure 1A) were most impacted for orthopedic surgery and otolaryngology, while outpatient volumes (Fig 1B) were most impacted for dental/oral surgery and otolaryngology, relatively elective specialties. Within otolaryngology, otology and general/pediatric otolaryngology suffered the largest decreases in monthly case volume in both the inpatient (71.5% and 45.5% decrease, respectively) and outpatient settings (57.7% and 50.3% decrease, respectively, Fig 1C) compared to 2019. Of outpatient cases, myringotomy and tonsillectomy/adenoidectomy volumes decreased the most (Fig 1D), by 71.5% and 51.2%, respectively, compared to 2019.

Hospitals in the Northeast and AMCs were associated with lower surgical volume during the pandemic compared to the prior year after adjusting for SVI and COVID-19 censuses. Logistic regression modeling of the outcome of having below-average annual volume compared to pre-pandemic volumes showed that AMCs were associated with over twice the odds of below-average volume in 2020–2021 (odds ratio [OR] 2.20, 95% confidence interval [CI] 1.16–4.21), with even greater decreases in the Northeast US (OR 3.19, 95% CI 1.61–6.47) after adjusting for bed-size, COVID-19 census, and inpatient SVI^4^ (Supplementary Table 2 in the online version of this article). The adjusted effect of SVI on case volume was minimal (OR 1.04, 95% CI 1.02–1.06), underscoring the importance of location and AMC status during the pandemic on case volume at the hospital system level.

These findings highlight the effect of the pandemic on surgical volume, particularly for highly ambulatory specialties such as otolaryngology. With the exception of oncologic and emergency procedures, these specialties broadly seek to improve quality of life through elective surgery and many of these operations were postponed. Academic and Northeast practices were the most affected, reflecting the Northeast as the frontline of the initial surge. Within otolaryngology, otologic and general/pediatric otolaryngology cases appeared to suffer the largest decreases in volume, supporting the theory that quality of life changes largely drove these findings. However, another possibility is that providers were encouraged to postpone upper airway cases due to anatomical concerns around COVID transmission.

These data provide a cautionary note for the provision of elective surgery amid the current emergence of the Omicron variant, the high likelihood of future variants, and severe staffing shortages nationwide. These data suggest that there is a pressing need for more dynamic and nuanced decision-making to allow elective surgical care to continue during COVID-19 surges. Ensuring bed capacity and resources to care for COVID patients is critical, but the needs of elective surgical patients (particularly in highly ambulatory specialties) must be addressed to avoid the surgical backlogs,^5^ postponed oncologic cases, and financial hurdles from depressed rates of elective surgery seen in the early pandemic.^2,6,7^

There are limitations here that should be acknowledged. Vizient data, while collected from across the country from AMCs and community hospitals, are not a statistically representative sample of American hospitals. Also, the data are analyzed at the specialty, hospital system, and department level, and conclusions should not be extrapolated to the patient level.

## CONCLUSION

These data show that otolaryngology and other ambulatory specialties were most impacted by the pandemic, and northeastern hospitals and AMCs had the highest risk of lower surgical volume. Within otolaryngology, otology and general/pediatric otolaryngology cases were affected most by the pandemic. The resulting financial pressures and surgical bottlenecks make it critical that we continually monitor the ancillary effects of COVID-19 across the healthcare industry and learn from the past, ensuring our ability to allocate resources judiciously in a field with limited capacity, finances, and human capital.

## Supplementary Material

Supplements
