# Supplementary material for: Comparison of subspecialty major surgical volume in the United States during the COVID-19 pandemic
Source: Laryngoscope. Author manuscript; Available in PMC 2023 Nov 1. (PMC9088461; doi:10.1002/lary.30066)
Supplement: Supplements [file NIHMS1793036-supplement-Supplements.docx]

## Supplement: Comparison of subspecialty major surgical volume in the United States during the COVID-19 pandemic

Anirudh Saraswathula, MD, MS^1^*^†^; Ernie Shippey, MS^2†^; Lee Ann Sprankle, MSN, RN, CPHQ^1^; Allen Kachalia, MD, JD^3^; Redonda G. Miller, MD, MBA^3^; Christine G. Gourin, MD, MPH^1^; C. Matthew Stewart, MD, PhD^1,3^

^†^These authors contributed equally to this work.

# Supplementary Methods

This study used data from the Vizient Clinical Data Base/Resource Manager™ (CDB) (Vizient, Inc. Irving, TX) and received human subjects research exemption from the Johns Hopkins University School of Medicine institutional review board. Data on surgical case volume and specialty was requested from hospital systems between January 1, 2019 and March 31, 2021. Cases were only included if the Current Procedural Terminology (CPT®) code was included in the Agency for Healthcare Research and Quality’s “narrowly defined surgery” procedure classification^1^ or if the ICD-10-PCS code was included in the “major diagnostic” or “major therapeutic” classification^2^. For inclusion, hospital systems needed to meet a threshold of 11 average surgical cases per month in 2019 and have reported case volume data for all 13 months between January 1, 2019 and March 31, 2021. Specialty-hospital system-month unit level data was excluded if there was missing data for 2019. Cases were filtered only to be included if the listed specialty of the billing physician was among the following: bariatric surgery, cardiothoracic surgery, colorectal surgery, dental/oral surgery, general OB/GYN, general surgery, gynecologic oncology, hand surgery, maternal and fetal medicine, neurosurgery, oncologic surgery, ophthalmology, orthopedic surgery, otolaryngology-head and neck surgery, pediatric surgery, plastic surgery, podiatry, thoracic surgery, transplantation, trauma surgery, urogynecology, urologic surgery, or vascular surgery. Inpatient or outpatient classification was done using Berenson-Eggers type of service codes.

For the calculation of specialty interruption index, volume from each speciality-hospital system-month was classified as “interrupted” if the observed:expected ratio of 2020-21 compared to 2019 volume was less than 70% (i.e., a greater than 30% decrease in volume for that specialty at that hospital system in that month). A weight was also calculated for each specialty-hospital system-month, the numerator being that month’s 2019 volume at the hospital system and the denominator the mean 2019 monthly volume for that specialty across all hospital systems. The percentage of “interrupted” months by specialty was calculated and multiplied by the weight to give greater weight to larger volume hospital systems.

Using the 299 hospital systems included in the outpatient volume analysis, a logistic regression was performed to evaluate for factors significantly associated with the outcome of below average annual outpatient surgical volume compared with the prior year. Assumptions of logistic regression were assessed, including evaluation for linearity in the logit, assessment of the residual plot, and calculation of the variance inflation factor to check for multi-collinearity. We included the following co-variables: bed-size, academic medical center status, geographic region, average annual COVID-19-positive patient census as a percentage of total inpatient census, and average inpatient CDC Social Vulnerability Index^3^, a composite metric developed by the CDC including poverty, lack of vehicle access, and crowded housing. Three hospital systems had missing data, one for geographic region and the other two for average annual COVID-19 positive cases as a percentage of total inpatient census. Since this represented only about 1% of the total data, these observations were excluded and a complete case analysis was done.

Data collation was performed using SAS v7.1.5 (SAS Institute Inc., Cary, NC, USA) and analysis with R v4.1.0 (R Foundation for Statistical Computing, Vienna, Austria) using the tidyverse^4^, and cowplot^5^ packages.

**Supplementary Table 1. Baseline hospital system** **characteristics stratified by annual outpatient surgical volume observed:expected ratio (SVI, social vulnerability index).**

|  | **Above average (*N* = 149)** | **Below average (*N* = 150)** | ***p*** |  |
| --- | --- | --- | --- | --- |
| Bed-size (≥250 beds, %) | 80 (53.7) | 108 (72.0) | 0.002 | ** |
| AMC (%) | 67 (45.0) | 100 (66.7) | <0.001 | *** |
| Region (%) |  |  | 0.003 | ** |
| Midwest | 53 (35.8) | 45 (30.0) |  |  |
| Northeast | 25 (16.9) | 51 (34.0) |  |  |
| South | 48 (32.4) | 30 (20.0) |  |  |
| West | 22 (14.9) | 24 (16.0) |  |  |
| Inpatient SVI (mean, SD) | 0.40 (0.12) | 0.45 (0.13) | 0.006 | ** |
| COVID-19 average census (mean, SD) | 0.07 (0.03) | 0.08 (0.03) | 0.845 |  |
| COVID-19 maximum census (mean, SD) | 0.20 (0.07) | 0.21 (0.10) | 0.493 |  |

Abbreviations: AMC (academic medical center); SVI (Social Vulnerability Index)

**Supplementary Table 2. Logistic regression model of risk factors for experiencing below-average annual outpatient surgical volume during the pandemic for 299 hospital systems.**

|  | **Coefficient** | **Odds ratio** | **95% CI** |  |
| --- | --- | --- | --- | --- |
| Intercept | 2.50 | – | – |  |
| Bed-size (≥250 beds) | 0.12 | 1.13 | 0.59-2.16 |  |
| AMC | 0.79 | 2.20 | 1.16-4.21 | * |
| Region |  |  |  |  |
| Midwest (ref.) | – | – | – |  |
| Northeast | 1.16 | 3.19 | 1.61-6.47 | ** |
| South | -0.41 | 0.66 | 0.35-1.26 |  |
| West | 0.23 | 1.26 | 0.59-2.69 |  |
| COVID-19 inpatient census | 0.02 | 1.02 | 0.93-1.12 |  |
| Inpatient SVI | 0.04 | 1.04 | 1.02-1.06 | *** |

Abbreviations: AMC (academic medical center); SVI (Social Vulnerability Index)

# Supplementary References

1. Healthcare Cost and Utilization Project (HCUP). Surgery Flags Software for Services and Procedures. Agency for Healthcare Research and Quality. Published May 2021. Accessed August 5, 2021. https://www.hcup-us.ahrq.gov/toolssoftware/surgeryflags_svcproc/surgeryflagssvc_proc.jsp

2. Healthcare Cost and Utilization Project (HCUP). Procedure Classes Refined for ICD-10-PCS. Agency for Healthcare Research and Quality. Published March 2021. Accessed August 13, 2021. https://www.hcup-us.ahrq.gov/toolssoftware/procedureicd10/procedure_icd10.jsp

3. Agency for Toxic Substances and Disease Registry. *CDC Social Vulnerability Index*. Centers for Disease Control and Prevention; 2021. https://www.atsdr.cdc.gov/placeandhealth/svi/fact_sheet/pdf/SVI_FactSheet_v10152020-H.pdf

4. Wickham H, Averick M, Bryan J, et al. Welcome to the Tidyverse. *Journal of Open Source Software*. 2019;4(43):1686. doi:10.21105/joss.01686

5. Wilke CO. *Cowplot: Streamlined Plot Theme and Plot Annotations for “Ggplot2.”*; 2020. Accessed August 13, 2021. https://CRAN.R-project.org/package=cowplot
